# Age as a Mediator of tDCS Effects on Pain: An Integrative Systematic Review and Meta-Analysis

**DOI:** 10.3389/fnhum.2020.568306

**Published:** 2020-10-28

**Authors:** Júlia Schirmer Saldanha, Maxciel Zortea, Iraci Lucena da Silva Torres, Felipe Fregni, Wolnei Caumo

**Affiliations:** ^1^Graduate Program in Medical Sciences, School of Medicine, Universidade Federal do Rio Grande do Sul (UFRGS), Porto Alegre, Brazil; ^2^Laboratory of Pain and Neuromodulation, Clinical Research Center, Hospital de Clínicas de Porto Alegre (HCPA), Porto Alegre, Brazil; ^3^Pharmacology of Pain and Neuromodulation: Pre-Clinical Investigations Research Group, Universidade Federal do Rio Grande Do Sul (UFRGS), Porto Alegre, Brazil; ^4^Laboratory of Neuromodulation and Center for Clinical Research Learning, Physics and Rehabilitation Department, Spaulding Rehabilitation Hospital, Boston, MA, United States; ^5^Pain and Palliative Care Service, Hospital de Clínicas de Porto Alegre (HCPA), Porto Alegre, Brazil

**Keywords:** adolescent, elderly, tDCS, pain, pain threshold, DLPFC, M1, meta-analysis

## Abstract

**Introduction:** The transcranial direct current stimulation (tDCS) is a neuromodulatory technique with the potential to decrease pain scores and to improve chronic pain treatment. Although age is an essential factor that might impact the tDCS effect, most studies are solely conducted in adults. Therefore, the age limitation presents a critical research gap in this field and can be shown by only a handful of studies that have included other age groups. To examine the evidence upon the tDCS effect on pain scores on children, adolescents, or elderly, and indirectly, to infer the age-dependent impact on tDCS effects, we conducted a systematic review and meta-analysis.

**Methods:** A systematic review searching the following databases: PubMed, EMBASE, and Science Direct using the following search terms adapted according to MeSh or Entree: [(“Adolescent” OR “Children” OR “Elderly”) AND (“tDCS”) AND (“Pain” OR “Pain threshold”) AND (“dorsolateral prefrontal cortex” OR “Motor cortex)] up to April 20th, 2020. We retrieved 228 articles, 13 were included in the systematic review, and five studies with elderly subjects that had their outcomes assessed by pain score or pain threshold were included in the meta-analysis.

**Results:** For the analysis of pain score, 96 individuals received active stimulation, and we found a favorable effect for active tDCS to reduce pain score compared to sham (*P* = 0.002). The standardized difference was −0.76 (CI 95% = −1.24 to −0.28). For the pain threshold, the analysis showed no significant difference between active and sham tDCS. We reviewed two studies with adolescents: one study using anodal tDCS over the prefrontal cortex reported a reduction in pain scores. However, the second study reported an increase in pain sensitivity for the dorsolateral prefrontal cortex (DLPFC) stimulation.

**Conclusion:** Our findings suggest tDCS may reduce pain levels in the elderly group. Nevertheless, the small number of studies included in this review—and the considerable heterogeneity for clinical conditions and protocols of stimulation present—limits the support of tDCS use for pain treatment in elderly people. Larger studies on the tDCS effect on pain are needed to be conducted in elderly and adolescents, also evaluating different montages and electrical current intensity.

## Introduction

Chronic pain is conceptually a process of maladaptive neuroplasticity by an imbalance in the excitability and inhibition in the pain processing pathways, including the cortical anatomical changes and the dysfunction in the processing as assessed by functional connectivity (Peyron and Fauchon, 2019). In contrast, the main mechanism of acute pain is tissue injury. This is a critical point that should be considered as part of the diagnostic criteria besides continuous or recurrent pain for more than 3 months (Raja et al., 2020). According to a survey conducted in 2016, over 20% of American adults suffered from chronic pain, and 8% presented high-impact pain, which is classified when pain is associated with limiting life or work activities (Dahlhamer et al., 2018). In elderly people, chronic pain is a major health issue. The prevalence of chronic pain among elderly Americans, between the ages of 65 and 85, is estimated at 27%, and over 85 years old, it reaches 33% (Dahlhamer et al., 2018). Chronic pain for elders is also the most common risk factor for disability (Melzer et al., 2005; Covinsky et al., 2009), due to daily living activities impairment and psychosocial problems related to fear of movement (Meier et al., 2016), depression (Casten et al., 1995) and reduced quality of life (Hopman-Rock et al., 1997). Besides, chronic pain among children and adolescents is also concerning, considering it may reach 44% prevalence in some countries (Gobina et al., 2019; Bondesson et al., 2020). The presence of chronic pain in this age group has a negative impact on quality of life, damaging the social, recreational, and academic domains, also being a major cause of absenteeism in children and adolescents. Chronic pain is associated with increased school abstention (Groenewald et al., 2019), and, as shown by prospective cohort studies, chronic pain in adolescents was associated with lower education levels in adulthood, worse career positioning, and other social impacts such as early parenthood and a worse quality in affective relationships (Murray et al., 2020).

On the treatment side, chronic pain is a challenging condition, with low levels of pharmacological therapeutic success (Moore et al., 2013) and risk of opioid dependence (Florence et al., 2016). More than 20% of children and adolescents with chronic pain receive opioid prescriptions, and 25% receive a prescription of two to four classes of drugs for pharmacological treatment (Gmuca et al., 2019). In this scenario, transcranial direct current stimulation (tDCS) gain major importance and appears as a promising therapeutic alternative based on the results found in the treatment of chronic pain syndromes in adults (Zortea et al., 2019). However, the tDCS effect has considerable interindividual variability between subjects (Ridding and Ziemann, 2010), such variability can account for over 50% of the tDCS effect over motor evoked potential (López-Alonso et al., 2014).

Moreover, the data that supports the use of tDCS on chronic pain syndromes was mostly reported within adults between the ages of 18 and 60 years, and chronological age has been pointed out as a relevant factor for inter-subject variability of the tDCS effect (Ridding and Ziemann, 2010). This aspect is supported by neurobiological mechanisms involved in the tDCS effect which depend on the personal propensity for plasticity induction. This propensity tends to be more significant at a younger age, and it decreases during life with a lower tendency to occur in older age (Ridding and Ziemann, 2010; Freitas et al., 2013). Moreover, the ability to induce plasticity with the use of neuromodulation techniques was previously shown to be reduced with age (Muller-Dahlhaus et al., 2008; Fathi et al., 2010).

The tDCS modulates cortical excitability with low-intensity continuous electric currents applied via electrodes placed on the scalp (Nitsche and Paulus, 2000; Nitsche et al., 2008). Anodal(a)—tDCS—enhances cortical excitability, while the cathodal tDCS decreases the excitability on respective target areas. According to animal models, the cortical excitability changes by a direct current occurring due to the modification of membrane potential of targeted neurons (Bindman et al., 1964; Purpura and McMurtry, 1965). Blockers of sodium channels (e.g., carbamazepine) and calcium channels (e.g., flunarizine), when used to assess the tDCS effect on the membrane potential, eliminate the standard increase in cortical excitability presented by anodal stimulation (Nitsche et al., 2003). Presumably, because these drugs cause hyperpolarization of neurons as they render sodium and calcium channels inactive (Stagg and Nitsche, 2011). Collectively, these results indicate that the tDCS effects include the modulation of neuronal membrane potential. In terms of spatial effect, the tDCS induces changes around the electrodes' region, but tDCS can also alter broad areas across the cortex, not only on the target area (Turi et al., 2012). Such results were found on human studies using electric current flow models and the function of nuclear magnetic resonance (fMRI) (Saiote et al., 2013).

Further evidence suggests that tDCS modulates synaptic activity by neurotransmitters. Human neuroimage studies using magnetic resonance spectroscopy (MRS) from analysis targeting specific neurotransmitter receptors found that anodal stimulation inhibits the GABA-ergic system (Liebetanz et al., 2002; Nitsche et al., 2004; Stagg et al., 2009; Stagg and Nitsche, 2011). While Citalopram, a serotonin reuptake inhibitor, reversed the inhibitory effect from a cathodal stimulation, and it enhanced and prolonged the excitatory effect for a-tDCS (Liebetanz et al., 2002; Medeiros et al., 2012). Through promoting or inhibiting cortical excitability, tDCS also elicits effects on cortical excitability that persist after the stimulation period, which occurs through synaptic plasticity mechanisms resembling the characteristics of long-term potentiation (LTP) or long-term depression (LTD) of glutamatergic synapses (Nitsche et al., 2008). Blockers of glutamate receptors NMDA abolish both excitatory and inhibitory aftereffects of tDCS. Therefore, the decreased ability to generate LTP found on elderly people (Barnes et al., 2000) can influence this neuromodulatory technique effectiveness. Moreover, the reduction in brain volume associated with senescence causes an increase in the distance between the brain cortex and the tDCS electrodes positioned over the scalp (Resnick et al., 2003), which can have, as a consequence, a lower electric field peak under the tDCS electrode for elderly (Thomas et al., 2018).

In contrast, studies have shown that children compared to adults with the same tDCS parameters have a peak electric field and current density at least 1.5 times greater than the one found in adults. This phenomenon is associated with a thinner skull bone and a higher volume of cerebrospinal fluid (Minhas et al., 2013). The impact of the skull thickness was demonstrated in the spatial dispersion model of electric current. The electric field was more potent in regions with thinner bone. Besides that, a regression model demonstrated that the cerebrospinal fluid's thickness and the depth of the cortical gyres also influence the distribution of the electric field (Opitz et al., 2015). Moreover, children and adolescents undergo a maturational physiological process that is comprised of synaptic selection and myelinization. This process is active until early adulthood and is associated with a higher plasticity basal status (Sowell et al., 2004). Finally, neurophysiological measuring showed that intracortical inhibition increases with age (Croarkin et al., 2014), and children and adolescents have a lower level of intracortical inhibition (Mall et al., 2004), which could turn them more susceptible to the tDCS effect.

Nevertheless, the maturation and senescence process, which occurs to children, adolescents, and the elderly, respectively, are non-linear phenomena (Sowell et al., 2004). Maturation begins at the parietal cortex, more specifically at the primary somatosensory cortex, and then progresses rostrally to the frontal and caudal cortex and laterally to the occipital, parietal and temporal cortex. The dorsolateral prefrontal cortex (DLPFC) is the last prefrontal area to undergo a maturation process, which occurs only in late adolescence (Gogtay et al., 2004). The senescence process occurs with a reduction in cortical volume due to a decrease in neuronal size and the consequent loss of the amount of gray matter, but also a loss of white matter volume is present (Good et al., 2001). In similarity with the maturation process, senescence is also a non-linear phenomenon, and the most considerable volume losses occur in frontoparietal lobes, while the occipital and temporal lobes have shown smaller volume reductions (Resnick et al., 2003). Therefore, due to localized differences in a non-linear process, the montage of tDCS and the electrical current intensity can elicit different effects for distinct cortical areas according to age across life. Thus, additional studies are needed to explore this issue regarding pain, since the two primary sites for the transcranial neuromodulation, nominally M1 and DLPFC, are likely affected differently by this neuromodulatory technique across life.

Presumably, based on plasticity induction, a-tDCS over the left DLPFC and M1 has been shown to decrease pain levels in chronic pain patients and to increase pain threshold in healthy subjects (Vaseghi et al., 2014; Zortea et al., 2019). Anodic stimulation over M1 seems to modulate the pain threshold and pain level by inhibiting thalamic nuclei's activity. The lateral thalamic nucleus targets spinal nociceptive afferent input from lateral thalamic nuclei responsible for sensory-discriminative aspects of pain stimulus (García-Larrea et al., 1999). The DLPFC is one of the most common active areas during pain episodes (Apkarian et al., 2005) and is essential in the decision process related to pain. The prefrontal cortex is part of the network of connections of the descending modulatory system of pain. It seems to modulate structures involved in the emotional perception of pain, including the medial prefrontal cortex, anterior insular cortex, anterior cingulate cortex, and bilateral amygdala. These areas are connected to structures such as the nucleus raphe magnus, periaqueductal gray matter, and the middle frontal gyrus (Schweinhardt and Bushnell, 2010; McMahon et al., 2013). Prefrontal cortex stimulation can also act by modulating pain correlated to such as anxiety, depression, and unpleasant sensation over a painful stimulus (Nitsche et al., 2009). Anodal stimulation over the left DLPFC can reduce the valence for negative emotional images (Pena-Gomez et al., 2011), also improving the valence of positive emotional facial expressions (Nitsche et al., 2012).

### Transcranial Direct Current Stimulation: Technical Factors and Neuroplasticity

The tDCS effects are influenced by the area where the electrode is applied (e.g., M1, DLPFC), anatomic aspects and factors related to neuroplasticity state-dependent (Ridding and Ziemann, 2010). Besides the discussed age impact on the tDCS effect, several other factors can be involved in the non-linear response to tDCS and the interindividual variability. Among them, there are factors related to neuroplasticity, such as genetics polymorphisms [e.g., brain-derived neurotrophic factor (BDNF) Val66Met, and Catechol-O-Metil Transferase (COMT) Val158Met], time of day, psychotropic drugs, exercise, circadian typology, cognitive processes, and sex. Regarding sex, women appeared to be more responsive to tDCS. Possibly an impact depends on estrogen levels. Women also showed a higher medial prefrontal activation under nociceptive stimuli (Gupta et al., 2017) and a higher inhibitory function of the descending pain modulating system compared to males (Gasparin et al., 2020). Additionally, there is vast literature related to sex differences in pain sensitivity (Bartley and Fillingim, 2013). Also, regarding the tDCS effect, women were shown to develop a longer-lasting LTD effect than men (Kuo et al., 2006).

Also, current intensity can be a moderator concerning age, since the tDCS effect results from the interaction of factors, including the stimulus (intensity, area of stimulation, cathodic or anodic, etc.) and neuroplasticity state. However, the previous literature related to the tDCS dose is mixed and suggests that it is not linear (Monte-Silva et al., 2013). It is plausible that at least part of this explanation can be related to tDCS electrical current intensity. However, it is crucial to consider that the tDCS stimulus might facilitate or inhibit synaptic transmission increasing or decreasing the frequency of action potentials in endogenous neuronal firing since it does not generate action potentials *per se*. Based on this rationale, we can comprehend why the literature is contentious regarding this point. A previous meta-analysis found that the tDCS higher dose was associated with better outcomes in patients with acute major depressive episodes (Brunoni et al., 2016). In contrast, a sham-controlled trial using a higher dose (2.5 mA) yielded non-significant findings (Loo et al., 2018). Other studies that applied tDCS over the motor cortex concluded that enhancement of tDCS dose did not necessarily increase the effects of stimulation, but might shift the direction of excitability alterations (Batsikadze et al., 2013; Monte-Silva et al., 2013). Overall, even though evidence to this moment is not conclusive, it seems to indicate that the variation of electrical current intensity certainly should be addressed when aiming to understand the differences on the tDCS effect.

**Figure 1:**
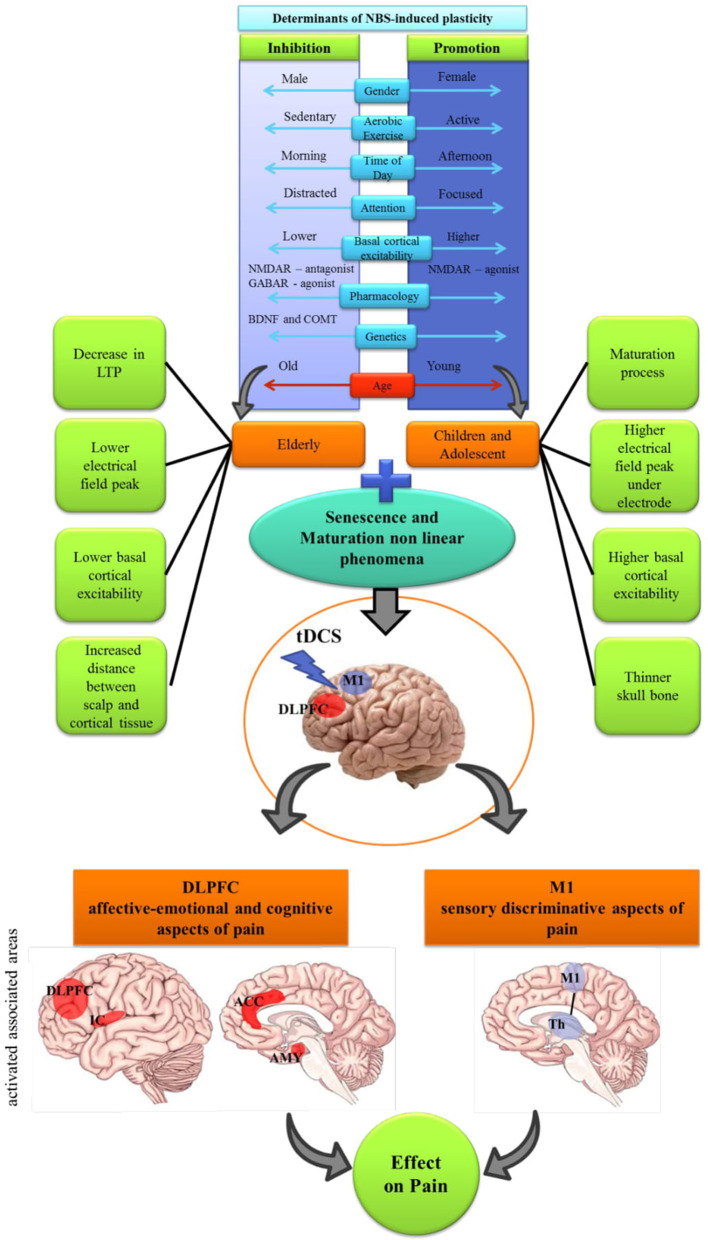
Schematic diagram demonstrating the integration of non-invasive brain stimulation (NBS) induced plasticity determinants. The non-linear feature of maturation and senescence processes should be considered combined with the M1 and DLPFC cortical association and different aspects of pain modulation. Core factors that influences proneness to LTP and LTD (e.g. sex, maturation of system; genetics); time of day; attention; pharmacology; etc, and technical features of the tDCS: area of stimulation (M1 and DLPFC); duration of stimulation the intensity of the electric current, influence on the effect of tDCS. IC, Insular Cortex; ACC, anterior cingulate cortex; AMY, amygdala; Th, thalamus.

Although tDCS has been gaining ground in the field of pain studies, and the evidence is growing in its favor, essential gaps remain regarding the understanding of mediating factors of neuroplasticity and how much they can modify the response ETCC, particularly in assembly and stimulation. Age has had a central role among these sets of factors that can permeate interpersonal variability in the effects of tDCS. The relationship between these factors involved in the neuroplasticity and the technical aspects is shown in Figure 1.

Given the emerging importance of tDCS as a potential treatment for chronic pain conditions, we have reviewed the current understanding of tDCS for pain in less studied age groups: children, adolescents, and elderly people. The goal was to compile and find evidence on how to treat pain and improve its correlated symptoms among the two most frequent montages of tDCS: M1 and DLPFC. This review intended to answer the following questions: (i) Can tDCS over M1 or DLPFC modulate pain level or pain threshold in children, adolescents, or elderly people differently? (ii) With this review, we have compiled data to generate evidence and discuss how age can impact these neuromodulatory techniques in distinct age groups for research and clinical contexts.

## Methodology of the Literature Review

This systematic review follows the PRISMA (Preferred Reporting Items for Systematic Reviews and Meta-analyses) guideline. There was no pre-published protocol.

### Search Strategy

To find relevant studies, we have conducted extensive literature research on the following databases: MEDLINE (from 1966), ScienceDirect (from 2006), EMBASE (from 1993). The resulting search terms according to MeSh or Entree were searched as follows: [(“Adolescent” OR “Children” OR “Elderly”) AND (“tDCS”) AND (“Pain” OR “Pain threshold”) AND (“dorsolateral prefrontal cortex” OR “Motor cortex)] up to April 20th, 2020 (complete search strategy for one database in Supplementary Material).

### Study Selection: Inclusion and Exclusion Criteria

Two authors reviewed and selected the included studies independently; if there were any disagreements, the subsequent decisions were discussed within a third-party reviewer. After retrieving the studies with the search strategy, the authors excluded duplicates, and the analyses were assessed within the inclusion criteria. The full text was evaluated if the study was potentially eligible. The following information was extracted: sample size, age, tDCS intervention, area of stimulation, electric current intensity, stimulation time, outcomes evaluated within the study, significant findings, and effect size.

**Figure 2:**
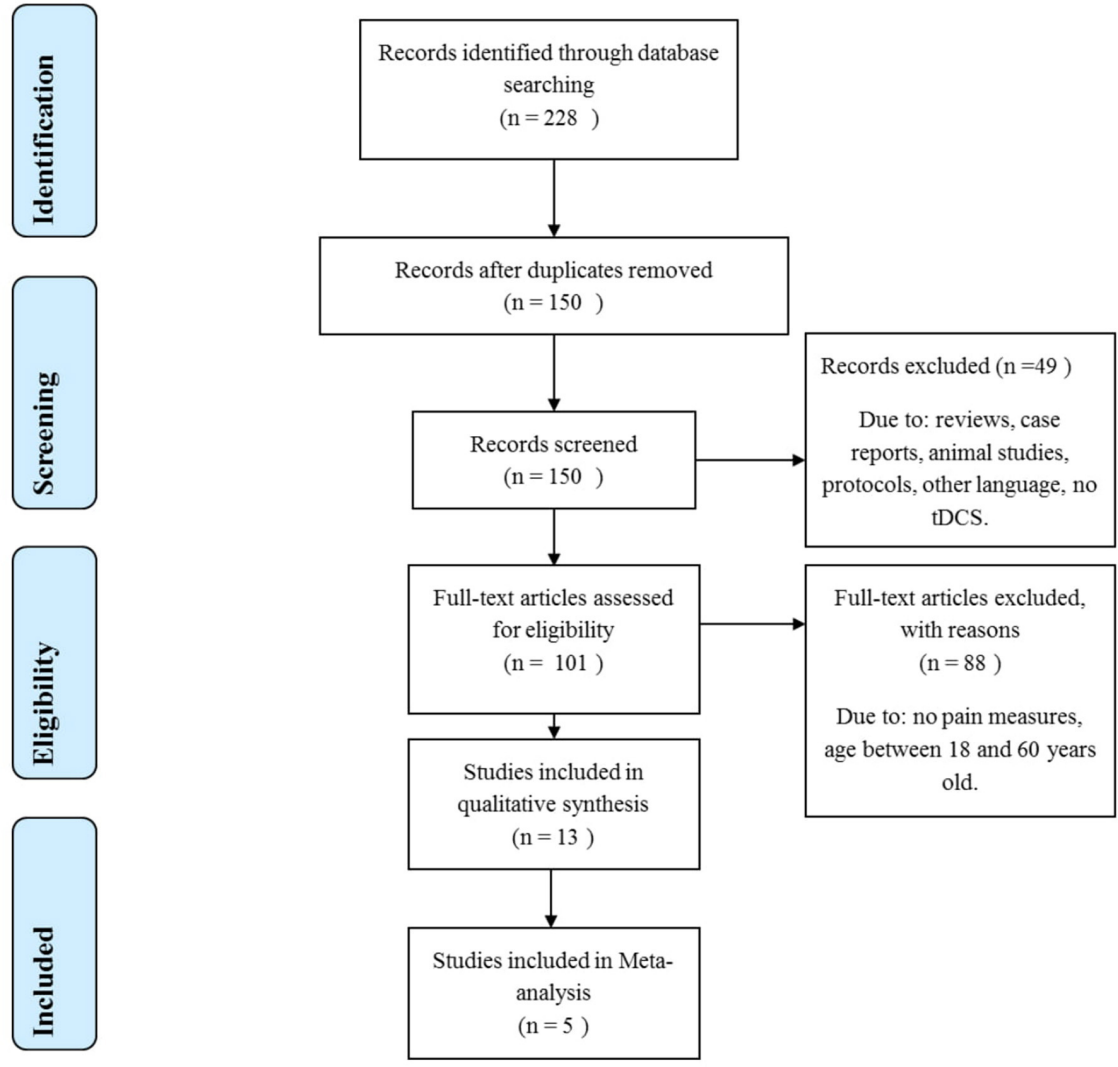
PRISMA flow for systematic review and meta-analysis.

The inclusion criteria were as follows: the sample was comprised of children, adolescents, or elderly individuals (60 years, or older) for the group of active tDCS. Studies used tDCS on the primary motor cortex (M1) or dorsolateral prefrontal cortex (DLPFC), either conventional tDCS, or high-definition (HD-tDCS). Studies should have a low risk of bias and outcome reporting visual analog scale (VAS) or numeric rating scale (NRS) pain scores or pain threshold to be included in the meta-analysis. We excluded studies with sample mean age ranging from 18 to 60 years old. Other exclusion criteria: languages other than English, Portuguese, or Spanish; not reporting pain or pain threshold as an outcome; studies using different types of stimulation rather than direct current; reviews and case studies; conference abstracts; study protocols. The fixed or random effect was applied according to heterogeneity, and the meta-analysis was performed by a standardized mean difference. The systematized research is presented in Figure 2.

### Risk of Bias Assessment

Two authors independently assessed the risk of bias according to the criteria from Cochrane guidelines (Higgins et al., 2011). Disagreements between the authors were solved by discussing such differences with the involvement of a third-party reviewer if necessary. The potential biases were classified as low risk (green plus signs), high risk (red minus signs), and unclear (yellow question mark) (Table 1). The features evaluated for bias: randomization (selection bias), allocation concealment (selection bias), blinding of participants and researchers (performance bias), incomplete outcome data (attrition bias), selective reporting (reporting bias); and other bias. Counterbalanced order for the different stimulation sessions and sham-controlled protocol were put into consideration regarding other biases categories.

**Table 1:** Assessment of risk of bias from the reviewed studies (*n* = 12).

**Study**	**Random Sequence generation**	**Allocation concealment**	**Blinding of subjects**	**Blinding of assessors**	**Incomplete outcome data**	**Selective report**	**Other bias**
Saldanha et al. (2020)	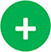	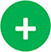	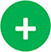	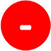	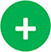	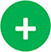	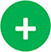
Lee et al. (2019)	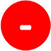	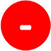	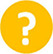	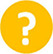	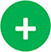	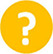	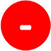
Ahn et al. (2019)	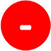	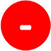	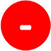	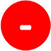	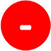	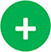	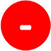
da Graca-Tarrago et al. (2019)	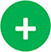	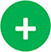	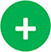	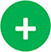	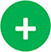	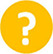	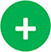
Deldar et al. (2019)	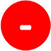	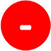	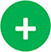	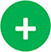	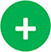	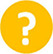	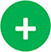
Borckardt et al. (2017)	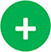	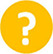	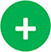	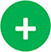	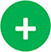	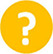	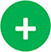
Harvey et al. (2017)	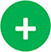	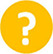	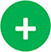	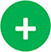	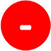	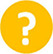	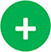
Ahn et al. (2017)	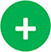	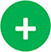	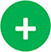	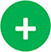	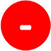	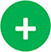	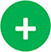
Concerto et al. (2016)	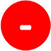	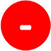	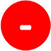	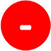	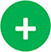	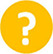	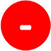
Hu et al. (2016)	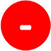	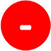	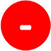	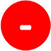	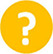	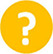	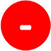
Borckardt et al. (2013)	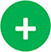	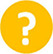	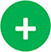	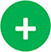	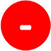	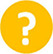	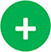
Kim et al. (2013)	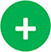	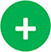	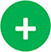	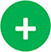	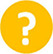	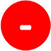	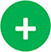
Pinchuk et al. (2013)	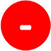	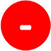	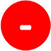	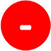	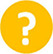	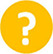	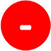

## Results

The search strategy identified 228 articles. After evaluating the title and abstract for duplicates and exclusion and inclusion criteria, 101 articles were selected for full-text evaluation, and 13 met the criteria to be included in this systematic review (Figure 2). The characteristics of the included studies are summarized in Table 2.

**Table 2:** Details of the studies included in the current systematic review (*n* = 13).

**Study**	**Design**	**Sample**	**Age**	***N***	**Intervention**	**Stimulation electrode**	**Reference electrode**	**Current intensity**	**Time (min)**	**N^**°**^ of sessions**	**Significant result**	**Cohen's D within**	**Cohen's D between**
**Elderly**													
Saldanha et al. (2020)	Cross-over	Healthy	63.8 (2.6)	9	a-tDCS	F3	FP2	2 mA	30	1	No significant effects of a-TDCS over M1 or DLPFC on heat pain threshold variation (delta) compared to sham	0.34	0.27
				9	a-tDCS	C3	FP2	2 mA	30	1		0.24	0.12
				10	s-tDCS	F3	FP2	Sham	30	1			
Lee et al. (2019)	Single arm	Neck and upper extremity non-inflammatory musculoskeletal pain	71.25 (4.54)¥	9	a-tDCS	F3	FP2	1, 2 mA	20	5 (sham) 5 (1 mA) 5 (2 mA)	↓ VAS pain score with 2 mA compared to baseline (within).	1 mA = 0.77 ^*^2 mA = 1.35	1 mA = 0.60 2 mA = 1.18
		Low back non-inflammatory musculoskeletal pain		22	a-tDCS	F3	FP2	1, 2 mA	20	5 (sham) 5 (1 mA) 5 (2 mA)	↓ VAS pain score with 1 mA and 2 mA compared to baseline (within)	^*^1 mA = 1.90 ^*^2 mA = 1.34	1 mA = 1.15 2 mA = 0.72
		Lower extremity non-inflammatory musculoskeletal pain		16	a-tDCS	F3	FP2	1, 2 mA	20	5 (sham) 5 (1 mA) 5 (2 mA)	No significant effects on VAS score from baseline	1 mA = 1.12 2 mA = 1.63	1 mA = 0.70 2 mA = 1.29
Ahn et al. (2019)	Single arm	Osteoarthritis	61.20 (7.23)	21	a-tDCS	M1 (side not specified)	SO	2 mA	20	10	↓ VAS pain score compared to baseline (within).	^*^Rosenthal's *R* = 0.62	NA
da Graca-Tarrago et al. (2019)	Parallel	Knee Osteoarthritis	66.0 (9.08)	15	a-tDCS + active EIMS	C3 or C4	SO	2 mA	30	5	↓ VAS pain score after treatment compared to s-tDCS + s-EIMS (between) and compared to baseline (within)	^*^1.86	^*^1.08
			64.1 (9.8)	15	a-tDCS + s-EIMS	C3 or C4	SO	2 mA	30	5	↓ VAS pain score compared to s-tDCS + s-EIMS (between) and compared to baseline (within)	^*^0.86	^*^0.36
			64.4 (6.02)	15	s-tDCS + active EIMS	C3 or C4	SO	Sham	30	5	↓ VAS pain score compared to s-tDCS + s-EIMS (between) and compared to baseline (within)	^*^1.22	^*^0.76
			63.87 (7.07)	15	s-tDCS + s-EIMS	C3 or C4	SO	Sham	30	5	↓ VAS pain score compared to baseline	^*^0.61	NA
Deldar et al. (2019)	Cross-over	Healthy	64.4 (4.4)	15	a-tDCS	F3	Right deltoid muscle	2 mA	22	2	a-tDCS significantly improved pain rating in NRS when associated to 2-back task condition compared to baseline No difference in pain for a-tDCS when compared to sham	^*^0.39	0.48
				15	s-tDCS	F3	Right deltoid muscle	Sham	22	2			
Borckardt et al. (2017)	Parallel	Post-operative pain	57.7 (11)	14	a-tDCS	C1 or C2	F4	2 mA	20	4	↑ hydromorphone use with a-tDCS over C1 or C2 compared to sham. No difference in post-operatory pain VAS between all groups.	A	a
			60.1 (6.7)	16	a-tDCS	F3	FPz	2 mA	20	4	↓ hydromorphone use with a-tDCS over F3 group compared to sham.	A	a
			62.5 (5.2)	15	a-tDCS	P3	FCz	2 mA	20	4	For a-tDCS over P3 there was no difference in hydromorphone use when compared to sham	a	a
			64.5 (8.8)	13	s-tDCS	C1, C2, or F3	F4 or FPZ	Sham	20	4		a	a
Harvey et al. (2017)													
	Parallel	Chronic pain	72 (6)	6	a-tDCS	C3 or C4	SO	2 mA	20	5	↓ VAS pain score (delta) from baseline to follow up assessment (7 days after the end of treatment) compared to sham (between)	^*^0.63	After treatment = 0.23 (lower score for sham)
			71 (8)	8	s-tDCS	C3 or C4	SO	Sham	20	5	↓ VAS pain score for the a-tDCS between baseline gathered VAS (7 days) and treatment gathered VAS (5 days) (within). No difference between active and sham on VAS for the last day of treatment.		Follow up (delta) = ^*^1.76
Ahn et al. (2017)	Parallel	Knee Osteoarthritis	60.6 (9.8)	20	a-tDCS	C3 or C4	SO	2 mA	20	5	↓ in NRS pain score (delta) for a-tDCS is significant different than sham for after the last session of tDCS treatment and for 3 week follow up.	a	After treatment = ^*^0.88 (delta) 3 week follow up = ^*^0.79 (delta)
			59.3 (8.6)	20	s-tDCS	C3 or C4	SO	Sham	20	5	NRS baseline level statically significant different between groups (a-tDCS higher baseline NRS)		
Concerto et al. (2016)	Single arm	Chronic plantar fasciitis	68.8 (3.3)	10	a-tDCS	C1 or C2	SO	2 mA	20	5	↓ VAS pain score compared to baseline to after the treatment and to 4 weeks follow-up	After treatment = ^*^2.464 week follow up = ^*^1.15	NA
Hu et al. (2016)	Single arm	Head and neck cancer	62.6 (5.0)	5	a-tDCS	C5	F4	2 mA	20	20	VAS of 2.94 and 1.59 were reported, respectively at baseline and at 1 week follow up No inferential statistics were presented.	0.02	NA
Borckardt et al. (2013)	Parallel	Post-operative pain	67 (9.1)	20	a-tDCS	C1 or C2	F4	2 mA	20	4	↓ hydromorphone use for a-tDCS compared to sham.	a	a
				20	s-tDCS	C1 or C2	F4	Sham	20	4	No difference in VAS between groups		
Kim et al. (2013)	Parallel	Diabetic Polineuropathy	59.6 (13)	20	a-tDCS	C3	SO	2 mA	20	5	↓ VAS pain score for a-tDCS over M1 compared to a-tDCS over DLPFC and sham after treatment. And for 4 week follow up VAS was lower than sham.	a	After treatment = ^*^1.72 4 week follow up = ^*^0.61
				15	s-tDCS	F3	Right deltoid muscle	Sham	22	2			
Borckardt et al. (2017)	Parallel	Post-operative pain	57.7 (11)	14	a-tDCS	C1 or C2	F4	2 mA	20	4	↑ hydromorphone use with a-tDCS over C1 or C2 compared to sham. No difference in post-operatory pain VAS between all groups.	A	a
			63.5 (8.7)	20	a-tDCS	F3	SO	2 mA	20	5	Pain pressure threshold variation from baseline to after treatment was higher for a-tDCS over M1 compared to sham and DLPFC.	a	After treatment = 0.50 4 week follow up = 0.27
			61.6 (10)	20	s-tDCS	C3	SO	Sham	20	5	VAS score after treatment or for 4 week follow up for a-tDCS over DLPFC was not different than sham.		
**Children/Adolescent**													
Saldanha et al. (2020)	Cross-over	Healthy	15.6 (0.5)	9	a-tDCS	F3	FP2	2 mA	30	1	↓ heat pain threshold (increase sensibility for pain) for the a-tDCS over DLPFC from baseline to after treatment (delta), significant different form a-tDCS over M1 and sham.	0.47	^*^1.09 (delta)
				9	a-tDCS	C3	FP2	2 mA	30	1		0.38	0.29 (delta)
				10	s-tDCS	F3	FP2	Sham	30	1			
Pinchuk et al. (2013)	Retrospective	Secondary to mild head injury chronic headache	13.6 (2.5)	38	a-tDCS	Frontal pole (interhemispheric fissure)	Ipsilateral mastoid process	60 to 90 μA	30–45	5–9	↓ NRS score from baseline to after treatment considering both montages	^*^1.91	NA
				6	a-tDCS	Center of the forehead	2 cm higher than mastoid process of the motor non-dominant hemisphere	60 to 90 μA	30–45				

EIMS, intramuscular electrical stimulation; CBI, brief cognitive-behavioral intervention; SO, Contralateral supraorbital area; NA, not applicable; a, no data available to calculate Cohen's D for VAS;

* *effect size of statistically significant results reported by the studies; ¥ age presented as a mean for all groups*.

*Cohen's D between active and sham was evaluated for the first score after the last tDCS session*.

### Results: Qualitative Data

**Figure 3:**
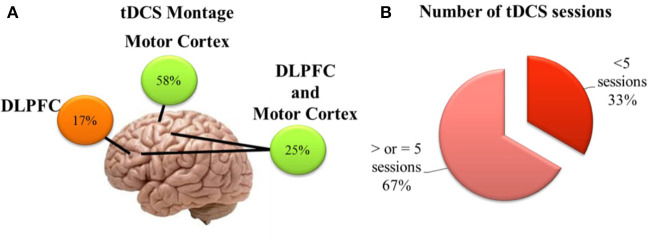
Brief schematic overview showing **(A)** position of electrodes on the scalp of the studies included in the systematic review; **(B)** Number of a-tDCS sessions in the protocols of the studies included in the systematic review.

The systematic review included 13 studies with data originated from 398 participants. Data regarding the montage, number of sessions, electric current intensity, and time of stimulation varied among studies. The total sample sizes of the studies varied from 5 to 60 subjects, several chronic pain conditions were included, as well as post-surgical pain and two studies evaluating the effect on pain threshold on healthy subjects. Seven studies applied a-tDCS on the Motor cortex, two studies used a prefrontal cortex stimulation, while three studies had an arm for a-tDCS over M1 and one over the DLPFC (Figure 3). One of the studies with adolescents applied the anodal tDCS over the frontal area or interhemispheric fissure. The number of tDCS sessions ranged from 2 to 20 stimulations each session. In one study, the a-tDCS was combined with intramuscular electrical stimulation. The tDCS current intensity was 2 mA for most of the trials with elderly participants; only one study evaluated a 1 mA current. For tests with adolescent subjects, the current intensity applied ranged from 60 μA to 2 mA stimulation. The simulation time was set to 20 min on nine studies. Other studies used 22, 30, or 45 min.

#### Studies Conducted With Elderly Subjects

**Figure 4:**
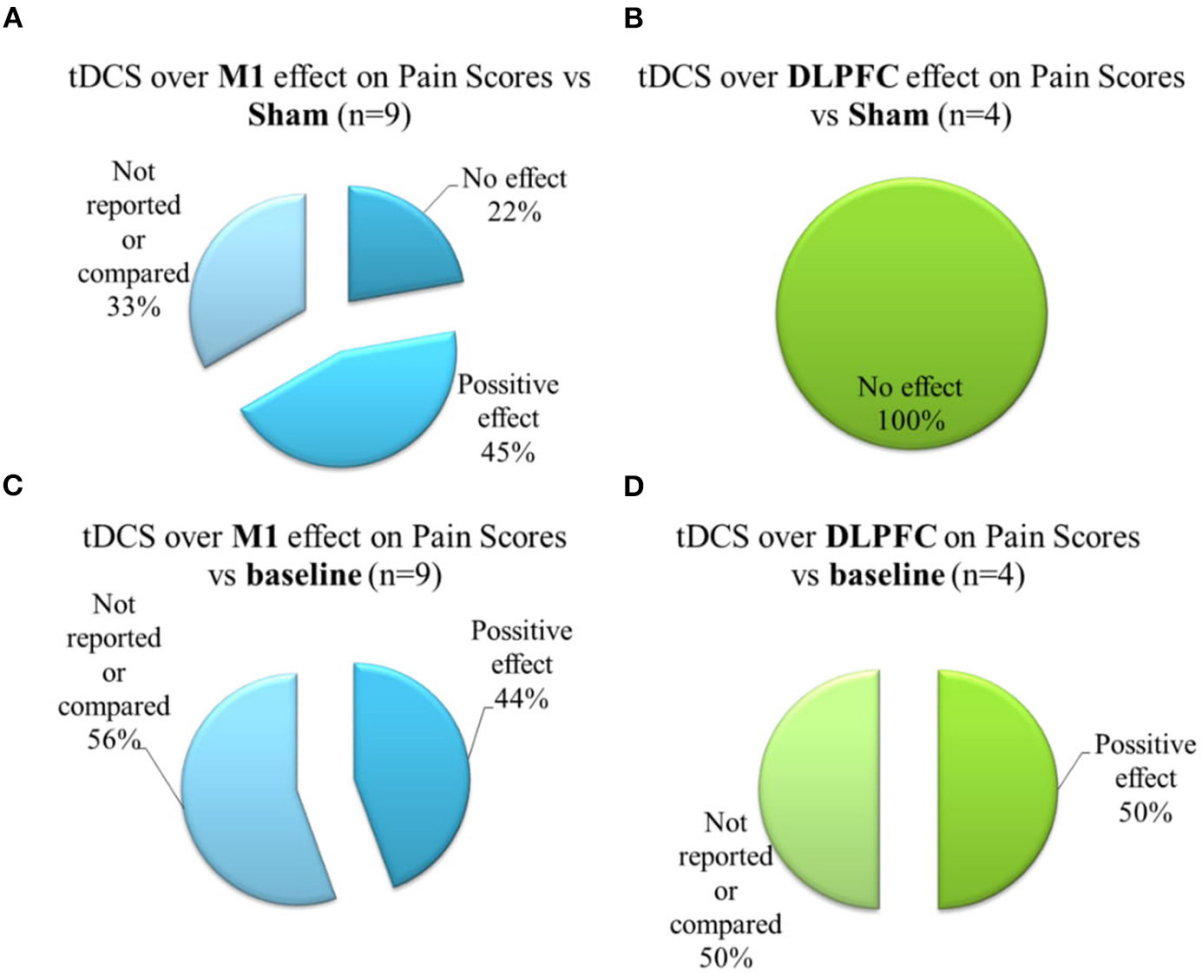
Brief schematic overview showing **(A)** Effects of a-tDCS on the M1 compared to sham-group on pain scores; **(B)** Effects of a-tDCS on the DLPFC compared to sham-group on the pain scores; **(C)** Effects of a-tDCS on the M1 on pain scores, within the group, from baseline to treatment end; **(D)** Effects of a-tDCS on the DLPFC on pain scores, within the group, from baseline to treatment end. Positive effect signalizes a decrease on pain scores.

Seven sham-controlled studies applied a-tDCS over M1, and six evaluated pain scores using VAS or NRS. The studies that assessed the outcome by pain score, four of them presented a significant improvement for a-tDCS compared to sham (Figure 4A). The size effect varies from moderate to large (Kim et al., 2013; Ahn et al., 2017; da Graca-Tarrago et al., 2019). The study of da Graca-Tarrago et al. (2019) was a factorial trial with groups that received a-tDCS coupled with intramuscular electrical stimulation (EIMS). The combined therapy with a-tDCS and active EIMS was more effective on pain decrease compared to sham (Cohen's *D* = 1.15, a large effect size). The use of a-tDCS, coupled with sham EIMS, produced an effect statistical significance compared to sham tDCS and sham EIMS, but the effect size was smaller (Cohen's *D* = 0.37). The other two studies that used a-tDCS on the M1 in a sham-controlled design did not find a significant difference between a-tDCS and sham for pain scores (Borckardt et al., 2013, 2017). The study that evaluated the pain threshold also did not find any difference from sham (Saldanha et al., 2020).

Four studies reported comparisons between before and after treatment effects for a-tDCS on the M1 with a large size effect for a-tDCS (Concerto et al., 2016; Harvey et al., 2017; Ahn et al., 2019; da Graca-Tarrago et al., 2019) (Figure 4C). Noteworthy among the studies that also had a sham group, da Graca-Tarrago et al. (2019) reported a VAS baseline reduction for active tDCS, but also for sham. Also, for M1 montage, three studies presented follow-up effects with a significant difference between a-tDCS and sham for 1, 3, and 4 weeks follow up with a large effect size of 1.79, 0.79, and 0.61, respectively (Kim et al., 2013; Ahn et al., 2017; Harvey et al., 2017).

Five studies applied a-tDCS over DLPFC, four of them were controlled with sham, and one of them evaluated the tDCS effect on pain scores in single-arm design. None of these studies reported a significant effect of a-tDCS stimulation compared to sham on pain scores (Figure 4B) or pain threshold. However, two studies reported a significant change in baseline pain (Figure 4D): Deldar et al. (2019) reported the effect comparing VAS before to after the application of a-tDCS when pain evaluation was conducted during a working memory task test. This difference presented a moderate effect size (0.39) (Deldar et al., 2019). Lee et al. (2019) found a significant difference for VAS pain score after a-tDCS over DLPFC was compared to the baseline with a large effect size. However, the study had no blinding or randomized order (Lee et al., 2019).

##### Evidence for Other Clinical Outcomes

Regarding analgesic use, earlier studies evaluated the tDCS effect in post-operative hydromorphone use (Borckardt et al., 2013, 2017). Borckardt et al. (2013) reported a decrease in hydromorphone use for a-tDCS over M1 when compared to sham. In the first study, they found a decrease in hydromorphone use when the a-tDCS was over M1 compared to sham (Borckardt et al., 2013). In contrast, in the second study, the same group found that the a-tDCS over M1 for the same montage increased the hydromorphone use compared to sham (Borckardt et al., 2017). The authors discussed the contradictory findings as being a consequence of the lack of blinding during the first study (Borckardt et al., 2013) and a more rigorous double-blind protocol for the second one (Borckardt et al., 2017). The other two studies in chronic pain reported a reduction in analgesic use with a-tDCS (Concerto et al., 2016) or with the EIMS, independently of a-tDCS or sham tDCS (da Graca-Tarrago et al., 2019).

Three studies with osteoarthritis samples reported the effect on the WOMAC scale, which is a self-administered questionnaire consisting of three subscales related to pain, stiffness, and impairments of physical function. Higher scores indicate worse pain, stiffness, and impairments of physical function. Ahn et al. (2019) and da Graca-Tarrago et al. (2019) reported an improvement in functional capacity from baseline (Ahn et al., 2019) compared to sham (da Graca-Tarrago et al., 2019). Ahn et al. (2017) did not find an a-tDCS effect on WOMAC. Three studies reported the effect on the McGill Pain Questionnaire (MPQ), which assessed multiple aspects of pain and its affective component. Two of these studies reported improvement in the MPQ from baseline for a-tDCS over M1 (Harvey et al., 2017; Ahn et al., 2019).

The three studies that applied a-tDCS over DLPFC also evaluated working memory performance. For Lee et al. (2019) and Saldanha et al. (2020), no effect was found, while for Deldar et al. (2019), a-tDCS reduced response time on n-back tasks when compared to baseline. Still, it was no different from sham, and no effect was found. Also, Lee et al. (2019) reported a reduced fear of pain and increased perceived self-efficacy, which translates as psychological confidence to perform specific activities. Both effects support the DLPFC connection to emotional and cognitive aspects of pain.

#### Studies With Adolescents

We included two studies with adolescents—one retrospective study with 44 adolescents with chronic headache related to mild head injuries treated with a-tDCS. The researchers have reported a decrease in the NRS score (range 0–10) of 3.5 points. They also found that the tDCS reduced the number of days of headaches per month and the span of the headache attacks. Moreover, 81% presented a complete improvement of headache episodes. Among them, 29% had a reduction of pain of at least 50% during 4.5 months after the treatment had ended (Pinchuk et al., 2013). The second study, a sham-controlled cross-over trial compared the heat pain threshold variation from before to after a-tDCS intervention on M1, DLPFC, and sham, and found an increase in pain perception (decreased pain threshold) for a-tDCS on the DLPFC compared to sham, with large effect size (Cohens'D 1.09) (Saldanha et al., 2020).

### Results: Meta-Analysis and Risk of Bias Assessment

We classified four studies with a high risk of bias due to the following reasons: open-label design, not randomized, no allocation concealment, or not presenting the blinding method description. A retrospective study from Pinchuk et al. (2013) presented the highest risk for bias, followed by open-label studies from Ahn et al. (2019), Concerto et al. (2016), and Hu et al. (2016). These four studies present a high potential for selection bias and performance bias. Another risk of bias was not being counterbalanced by the different number of stimulation sessions (Lee et al., 2019) or not being sham-controlled (Pinchuk et al., 2013; Concerto et al., 2016; Hu et al., 2016; Ahn et al., 2019). Also, most studies did not clearly describe the methodology used to compute the sample size for the primary outcome, and five studies were pilot studies. Three studies did not report on how they worked with the missing data from drop out participants.

We have selected five studies conducted with elderly participants for two meta-analyses. In the first analysis, we have included studies that reported the outcome using pain scores. For the secondary analysis, we also included studies that assessed the outcome utilizing the pain threshold. The included studies had a low risk of bias and we followed the Cochrane guidelines (Higgins et al., 2011), and used Review Manager 5 software to build forest plots (RevMan 5.3).

**Figure 5:**
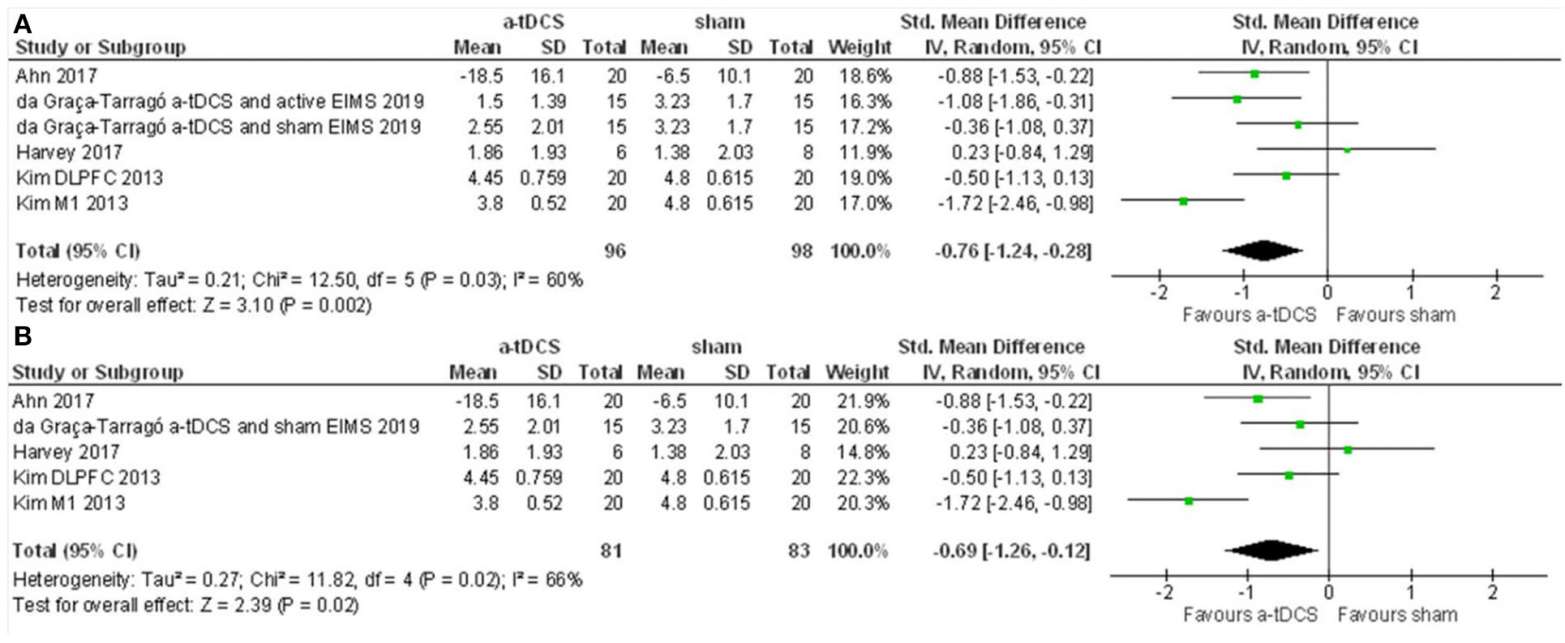
**(A)** Forest Plot on the effect of a-tDCS compared to sham for pain level (*n* = 4). **(B)** Sensitivity analysis for the pain level without the groups of trials combining active EIMS intervention (da Graca-Tarrago et al., 2019).

**Figure 6:**
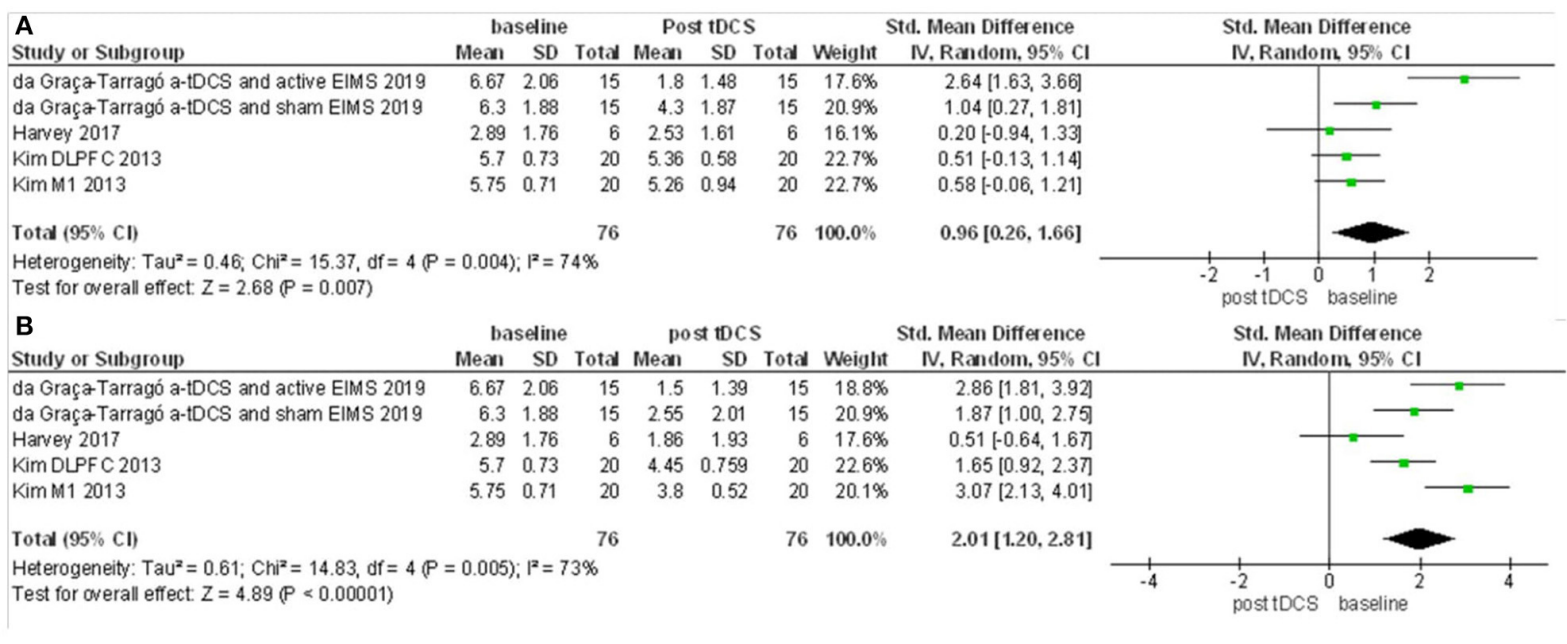
Forest Plot on the pain scores after a-tDCS compared to baseline. **(A)** Baseline compared to after first tDCS session. **(B)** Baseline compared to after five sessions tDCS treatment (*n* = 3).

The first meta-analysis evaluated outcomes for pain scores and included four studies and six intervention groups: four groups applied stimulation over M1, and two over the DLPFC. The data had considerable heterogeneity (I2 = 60%). Therefore, a random-effects model was applied. A total of 96 patients received active stimulation, and most of the studies favored active tDCS compared to sham tDCS on the improvement of pain score. The standardized mean difference was −0.76 (CI 95% = −1.24 to −0.28). We further performed a sensitivity analysis without the study with active EIMS. The effect size was reduced, but the difference had maintained a significant effect for a-tDCS compared to sham [−0.69 (−1.26 to −012)] (Figure 5). We compared the pain score from before and after one session of tDCS and after five sessions. Pain scores were reduced from baseline for either after a single session tDCS [0.96 (0.26 to 1.66)] and after five sessions [2.01 (1.20 to 2.81)] (Figure 6).

**Figure 7:**
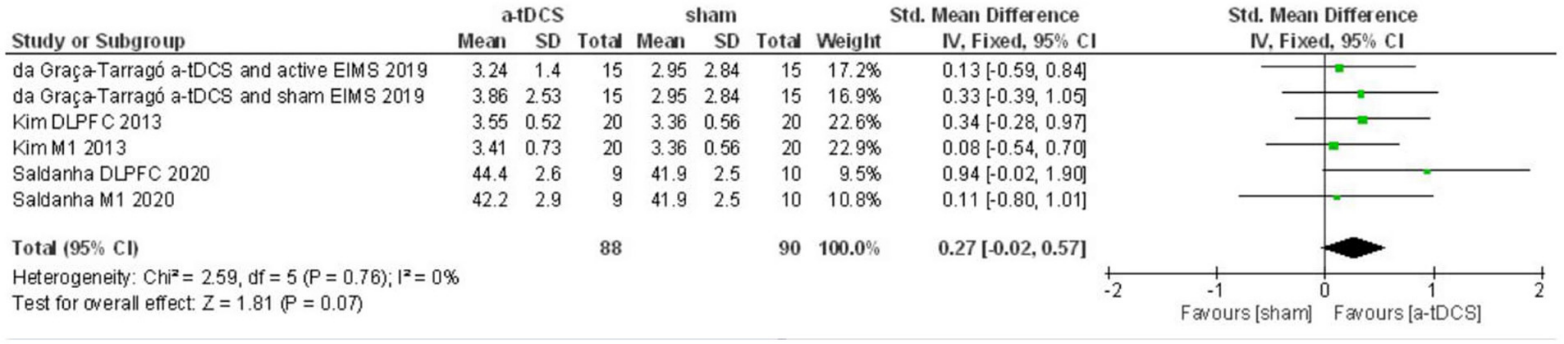
Forest Plot on the pain threshold after a-tDCS compared to sham.

For the second meta-analysis with pain threshold outcome, three studies were included, and six intervention groups were analyzed for both sites of stimulation. The data did not show heterogeneity in this condition (I2 = 0%). Thus, we applied the fixed effects model. A total of 88 patients received active stimulation. The effect of a-tDCS was not different from sham for pain threshold (ES = 0.27, *P* = 0.07, Figure 7).

## Discussion

In our systematic review and meta-analysis, we compiled data regarding the tDCS effect on pain level and pain threshold on elderly patients. We also collect data from two studies with adolescents. Our data intends to present an overview of the tDCS effect on these age groups. Our review on three relevant databases has revealed that only a few studies included these age groups. For this review, following the Cochrane guidelines, at least half of the studies reached good methodological quality, indicating that reported results for these studies are prone to a small bias effect.

### Studies With Elderly Population

For the included studies with the sham comparison group, a significant difference of a-tDCS compared to sham was reported in four out of eight studies with the elderly population. All the studies with significant differences also presented a large effect size and included patients with knee osteoarthritis (Ahn et al., 2017; da Graca-Tarrago et al., 2019), diabetic polyneuropathy (Kim et al., 2013), and also a combination of several clinical conditions were included in one study (Harvey et al., 2017). These chronic pain conditions included the following: osteoarthritis, sprained shoulder, chronic low back pain, cervical injury, shoulder tendinitis, polymyalgia rheumatic, sciatica, and unspecific leg pain (Harvey et al., 2017). The study with multiple chronic pain conditions from Harvey et al. (2017) did not find a significant difference in pain measures for the first evaluation after the treatment ended when compared to sham. However, they found a difference in pain scores between a-tDCS and sham groups in the 7 days follow-up. The variability of clinical conditions and the smaller sample size may explain a possible type II error in the first assessment. An alternative explanation for the effect in the follow-up may be the neuroplasticity changes induced by the a-tDCS. Our meta-analysis included these four studies that were evaluated as having high methodological quality. The cumulative meta-analysis effect indicated a moderate effect size of 0.76. This effect was kept significant after the sensitivity analysis when we removed the study of those groups that had received active peripheral stimulation from da Graca-Tarrago et al. (2019).

The meta-analysis for the modification of pain levels, when compared to baseline, presented a moderate effect size for both: one session of tDCS and five sessions. However, this effect size seems to increase across the repetitive course, with a tendency to be higher after five sessions of a-tDCS. Moreover, the tDCS effect on pain level might be sustained, since three studies reported a follow up persistent significant difference from a-tDCS and sham over 1 week (Harvey et al., 2017), 3 weeks (Ahn et al., 2017), and 4 weeks (Concerto et al., 2016). Further positive effects are outcomes that evaluate disability or affective pain components as well as decrease analgesic use.

The studies included in this review and in the meta-analysis suggest that a-tDCS for elderly subjects might be effective in decreasing pain levels. However, only tDCS stimulation over M1 had a different effect from sham. Two studies applied anodal tDCS over DLPFC and had a significant decrease in pain level from baseline, but no effect when compared to sham (Deldar et al., 2019; Lee et al., 2019). The results for elderly participants show similarities with previous studies with non-elderly adults for the tDCS effect over M1 for pain levels. Two previous meta-analyses evaluated anodal tDCS over M1 effect for pain threshold and pain level without setting age as an inclusion criterion (Vaseghi et al., 2014; Zortea et al., 2019). Combined studies included in both meta-analyses had participants' mean age ranging from 22 to 63.5 years old, and only 7% of the studies had a mean age above 60. Both meta-analyses reported that a-tDCS was effective in reducing pain levels with a moderate effect size for chronic pain. Moreover, Vaseghi et al. (2014) also presented an increase in pain threshold in healthy subjects for a-tDCS over M1 with a pooled mean difference of 22.19% compared to baseline and 12.57% compared to sham (Vaseghi et al., 2014).

Nevertheless, the results with elderly subjects differ from younger adults since both previous meta-analyses reported a significant effect on reducing pain levels for a-tDCS over DLPFC compared to sham. The meta-analysis conducted by Vaseghi et al. (2014) found a larger magnitude with a-tDCS over DLPFC compared to sham on pain level than over the M1 with a mean difference of 15.79 and 9.59, respectively. Zortea et al. (2019) also found a significant effect of DLPFC on pain levels with a moderate effect size (active compared to sham, Standard mean difference [SMD] = 0.54), although slightly smaller than M1 effect size when compared to sham (SMD = 0.68).

According to our meta-analysis, the impact of tDCS on the M1 compared to sham on pain levels on elderly patients pointed out in the same direction to the effect found on younger adults. This argument is supported by neuroimaging studies, suggesting that anodal tDCS promotes plastic alteration in the cortical areas of elderly subjects. Data from the fMRI study of GABA levels showed that neoplastic changes were present in the elderly brain. After a single session of a-tDCS over M1, they found reduced GABA levels in certain brain areas. According to the authors, this result reflects the tDCS effect on brain chemistry (Resnick et al., 2003). Moreover, one study reported the comparison of tDCS effect over M1 between young and older adults for motor evoked potential (MEP), as a measure of corticospinal excitability, and found no significant difference between the age groups (Fujiyama et al., 2014).

As discussed above, a-tDCS over DLPFC accounts for more marked differences between this review and meta-analysis and previous meta-analysis. The difference between tDCS over DLPFC might be related to a reduced number of studies on elderly groups. Combined with this perspective are the methodological differences in sample characteristics and the variety of aspects related to stimulation. A second reason could be related to the senescence process on elderly people, which can be more active in prefrontal areas than on the motor cortex (Resnick et al., 2003).

Noteworthy, chronic pain syndromes included in the meta-analysis from Vaseghi et al. (2014) and Zortea et al. (2019) are heterogeneous, as well as in the current review, which could impact on the a-tDCS effect. Chronic pain syndromes are associated with structural and functional brain alterations, which differ among conditions. In chronic musculoskeletal pain syndromes, it was reported a decreased gray matter volume in cortical areas associated with pain processing. Among them, the anterior cingulated cortex, the motor cortex, and the prefrontal cortex. Also, functional connectivity involving prefrontal cortex structures is modified (Coppieters et al., 2016). In contrast, a study with patients presenting peripheral neuropathic pain associated with diabetes showed decreased functional thalamocortical connectivity (Cauda et al., 2009). Thus, heterogeneity between clinical conditions should be taken carefully into account. Regarding the a-tDCS effect on different clinical syndromes, a study included in this review showed that the impact on a pain level of a-tDCS was distinct for different subgroups of non-inflammatory musculoskeletal pain, including neck and upper extremity pain, low back pain, and lower extremity pain. The a-tDCS was effective in improving the pain score only for the first two groups (Lee et al., 2019).

Finally, pain measures can also influence the result interpretation. Pain score ranges can vary in intensity, quality, and duration, according to the core diagnosis of disease, physiopathology, and correlate symptoms due to pain. In this meta-analysis, most studies assessed pain using unidimensional pain measures (i.e., numerical pain scale or visual analog scale), or pain threshold. Additionally, there are distinct pain conditions, including acute pain in a controlled experimental setting (Saldanha et al., 2020), post-surgical pain (Borckardt et al., 2013, 2017), and other chronic pain conditions (da Graca-Tarrago et al., 2019). For the pain assessment in an experimental acute pain model, when the target is pain intensity, these measures presented satisfactory psychometric properties. Thereby, these unidimensional measures do not assess other aspects correlated with chronic pain, such as sleep quality, humor, disability due to pain, etc. In comparison, the psychophysical pain measures give us neuropsychological insights about pain processing. That also helps us to understand the impact of TDCS on the dysfunction of pain pathways, for example, in pain perception (i.e., pain threshold) and the function of the descending pain modulatory system as assessed by the conditioned pain modulation test (Boggio et al., 2008; Reidler et al., 2012; Flood et al., 2016).

In contrast, psychophysical measures are not practical for widespread use in the bedside clinical setting, as VAS and NRS are widely used in the clinical setting due to their feasibility. Thus, different pain measures among the studies included in the meta-analysis can restrict these findings' general status. This way, further studies are needed to generate evidence on the tDCS effect within more representative samples. They should also target integrative approaches to comprehend its impact more extensively regarding pain as an illness and other to parameters that compromise patients' quality of life with chronic pain such as sleep quality, psychological symptoms, cognition, etc.

In this review, we have chosen to use the term “pain” to allow a broader search strategy, since there is scarce literature with children, adolescents, and elderly people. We've agreed that the tDCS effect should be individualized according to pain conditions. Particularly because chronic pain is conceptually a process of maladaptive neuroplasticity by an imbalance in the excitability and inhibition in the pain processing pathways. That also includes cortical anatomical changes and the dysfunction in the processing as assessed by functional connectivity. In contrast, the main mechanism of acute pain is tissue injury. This is a critical point in the interpretation of results to consider that the experience of pain cannot be reduced to activity in sensory pathways. For the tDCS applicability, either in the research and clinical setting, these aspects are relevant since the neuromodulatory response is likely neuroplasticity state-dependent, and this argument is also supported by the pain concept that defines it as a subjective experience that is influenced to varying degrees by biological, psychological, and social factors.

### Studies With Adolescents

For the adolescent's age group, one retrospective study included in the review found a positive effect of a-tDCS over the prefrontal cortex for headache treatment in adolescents. The second study was crossover-controlled in a sample of healthy females. The study evaluated the heat pain threshold after a single session tDCS, and the main result revealed that a-tDCS over DLPFC increases pain sensitivity. There are significant differences in the tDCS protocols between the studies related to the duration of stimulation, the intensity of the electric current, and the number of sessions. One of the studies has included chronic pain patients, and the other evaluated healthy participants. The foremost conclusion about the tDCS effect in adolescents is the lack of studies evaluating its impact on children and adolescents for pain levels. Chronic pain is a growing concern in these age groups, and it is associated with social and emotional burdens that can even impact adult life. Therefore, exploring new therapeutic options is a reason to justify that this subject is further investigated in controlled studies.

### Safety

The studies conducted with elderly subjects included in this review have not reported significant adverse effects. All the reported adverse effects were classified as mild according to the recommendations in the ICH guidelines (Baber, 1994; International Conference on Harmonisation, 2011). A mild adverse effect is a symptom that requires no medical treatment. The most reported adverse effects were itching, tingling, stinging, pins, and needles, burning sensation, all symptoms similar to tDCS previously reported most prevalent undesirable effects (Poreisz et al., 2007; Brunoni et al., 2011). In one of the studies included in this review, a participant dropped out of the study after reporting dizziness and sleep disturbances. However, the subject reported these adverse effects during the sham phase (Lee et al., 2019), while in another study, a participant dropped out due to headaches (Kim et al., 2013). The other studies included in the review reported that when adverse events were presented, they were mild (Hu et al., 2016; Ahn et al., 2017, 2019; Borckardt et al., 2017; da Graca-Tarrago et al., 2019; Saldanha et al., 2020).

Studies with adolescent participants also did not report major adverse effects. Saldanha et al. (2020) reported itching and sleepiness as the most prevalent undesirable effects. However, the active protocols applied over M1 and DLPFC were not different from sham in adverse effects incidence (Saldanha et al., 2020). These findings are supported by an extensive literature that has presented the adverse effects of most incidents in children and adolescents frequently classified as mild, such as itching, tingling, and headaches (Krishnan et al., 2015; Moliadze et al., 2015; Ciechanski and Kirton, 2017). Also, a recent study with more than 600 tDCS sessions on children and adolescents did not find severe adverse effects, being tingling and itching the most common adverse effects reported in 37% of tDCS sessions (Zewdie et al., 2020).

### Blinding

Blinding of the subjects that receive the treatment and of the evaluators is a major concern of randomized controlled studies using tDCS. Regarding the blinding of participants for tDCS use, using sham protocol has shown to be effective for a single session. However, questions arise for blinding when in use of higher current intensity and for multiple days' tDCS protocol. Moreover, the blinding of evaluators can be challenging due to scalp redness associated with active stimulation (Brunoni et al., 2012). Moreover, it is discussed that the use of a sham tDCS treatment applied at an intensity of 2 mA is not as effective as a blinding technique (O'Connell et al., 2012).

On the studies included in this meta-analysis that evaluated blinding effectiveness for 2 mA current intensity, they reported no difference in guessing between active and sham protocol from chance (Harvey et al., 2017; da Graca-Tarrago et al., 2019; Saldanha et al., 2020). The study conducted by Borckardt et al. (2017), that was not included in the meta-analysis also did not report any difference in guessing from participants between active and sham for 2 mA current intensity.

Therefore, for elderly subjects, 2 mA intensity blinding appears to be effective. Nevertheless, it is also noteworthy that blinding effectiveness was only accurately assessed in a fraction of the studies included in this review.

### Limitations

This review's primary limitation is the small number of articles and the critical heterogeneity among them. These limitations preclude us from drawing firm conclusions regarding the impact of age as a mediator of the tDCS effect. However, this is a relevant and emerging research field that can significantly affect pain treatment, given the prevalence of chronic pain increase among the elderly population and the lack of alternatives to improve the dysfunctional neuroplasticity that generates and sustain chronic pain. Additional aspects that prevent definitive conclusions are the risk of bias analysis and studies with small samples. Another factor that restricts the strength of the recommendation grade is the lack of accurate blinding assessment.

Furthermore, there is limited data on the possibilities to maintain the therapeutic effects over time. There is a substantial lack of literature that covers extended stimulation protocols taking into consideration the influence of age. In this review, we cannot explore the tDCS dose-effect according to age, since we found out that only two studies with adolescents (one observational study that applied current intensity of 60 μA and another study is a crossover trial, which used a current intensity of 2 mA in healthy individuals). Considering the limitations mentioned above, we have limited data to discuss the impact of electrical current delivery on the tDCS outcome effect according to age. Thus, more studies are needed before allowing us to draw any definitive conclusions about this topic.

### Future Perspectives

The international scientific community recognizes the importance of producing high-quality evidence of clinical efficacy regarding neuromodulatory techniques, including the tDCS. Aligned to this perspective, a recent meta-analysis points to benefits of tDCS usage in the treatment of many disorders, e.g., neuropathic pain, Parkinson's disease (motor), epilepsy, depression, schizophrenia, chronic and subacute stroke (motor without robotics), and post-stroke aphasia (Fregni et al., 2020). We can also include the primary chronic pain as a prototypical condition in this disease setting the fibromyalgia. Scientific studies support the impact of tDCS and its reliance upon protocol interaction with neuroplasticity state (Rozisky et al., 2016). The tDCS effect is dependent on neuroplasticity state because its action mechanism occurs through altering neuronal resting membrane potentials without inducing action potentials. Thus, if the goal is to facilitate the remapping or repairing in the brain's neuroplasticity, then we must employ an individualized protocol best suited for each patient. In this perspective, the tDCS can remap the dysfunction of neural networks.

There is growing evidence in favor of DCS use for pain treatment. However, a critical point that needs to be understood is the variability of response between subjects, even with the same syndrome diagnosis. Several different aspects need to be considered in further studies in order to generate consistent evidence. Thereby, it is possible to customize the model for stimulating neuroplasticity, considering age as a mediator of the response. Therefore, from this perspective, some points must be suggested: (i) Run trials with proper methodology and homogenous sample related to clinical diagnosis. (ii) Investigate distinct types of stimulation associated with cerebral area and different intensity of electric current and specific electrical current (i.e., continuous, random noise, or alternate). (iii) Assess neuroplasticity state using neuro markers, which are defined as traits or characteristics to customize treatment planning. Among them, neuropsychological measures, serum markers (i.e., BDNF) and genetic polymorphisms, etc. (iv) Investigate tDCS effects using integrative approaches, including the assessment of functional connectivity accompanied by clinical measures (i.e., disability, attention, anxiety, fear, and expectation, etc.). Additionally, it is essential to investigate the effect of combined interventions, such as online or offline, and the production of additional benefits or the system's overload and the production of a reverse effect. Thus, in an integrative view, we should tend toward precision medicine for prescribing treatments. Particularly in pain, even though the number of treatment targets has grown substantially, the clinical management is often an unsatisfactory journey for patients and clinicians. In sum, the tDCS presents promising properties in improving the complex network of delicate interconnected neurons and synapses conceptualized. This effect revolves around three main components: structural, electromagnetic, and neurochemical. These not only overlap each other, but they also work together in associating with the reorganization of structure, function, or connectivity involved in the dysfunctional processes underpinned to chronic pain and correlated symptoms.

## Conclusion

Our findings suggest the existence of a possible positive effect of a-tDCS in reducing pain on elderly subjects that received tDCS over M1. Regarding tDCS over the left DLPFC, a potential benefit on pain level was observed within the a-tDCS group. Also, the small number of studies and the heterogeneity among them preclude the generation of evidence supporting the impact of age as a mediator of the tDCS effect on pain measures in a generalizable way. Thus, they do not allow drawing more firm conclusions regarding the tDCS use to treat chronic pain or modulate pain threshold on elderly subjects. Therefore, further studies should be conducted, including distinct age groups, to better understand age impact on a-tDCS effects within pain in different clinical settings.

## Author Contributions

JS, MZ, IT, FF, and WC conceived and designed the study. JS, MZ, and WC participated in the data collection, performed the statistical analysis, and coordinated and drafted the manuscript. All authors contributed to the article and approved the submitted version.

## Conflict of Interest

The authors declare that the research was conducted in the absence of any commercial or financial relationships that could be construed as a potential conflict of interest.
