# Supplementary material for: Age as a Mediator of tDCS Effects on Pain: An Integrative Systematic Review and Meta-Analysis
Source: Front Hum Neurosci. 2020 Oct 28;14:568306. doi: 10.3389/fnhum.2020.568306 (PMC7654216; doi:10.3389/fnhum.2020.568306)
Supplement: Supplementary file 1 [file Data_Sheet_1.PDF]

SUPPLEMENTARY MATERIAL

Search strategy for EMBASE
[('aged'/exp OR 'aged' OR 'aged patient' OR 'aged people' OR 'aged person' OR 'aged subject' OR 'elderly' OR 'elderly patient' OR 'elderly people' OR 'elderly person' OR 'elderly subject' OR 'senior citizen' OR 'senium' OR OR 'adolescent'/exp OR 'adolescent' OR 'teenager' OR 'child'/exp OR 'child' OR 'children') AND ('transcranial direct current stimulation'/exp OR 'tdcs (transcranial direct current stimulation)' OR 'transcranial direct current stimulation') AND ('dorsolateral prefrontal cortex'/exp OR 'dorsolateral prefrontal cortex' OR 'motor cortex'/exp OR 'brain cortex, motor' OR 'brain motor cortex' OR 'cerebral cortex, motor' OR 'cortex, motor' OR 'motor brain cortex' OR 'motor cerebral cortex' OR 'motor cortex') AND ('pain'/exp OR 'acute pain' OR 'deep pain' OR 'lightning pain' OR 'nocturnal pain' OR 'pain' OR 'pain response' OR 'pain syndrome' OR 'treatment related pain' OR 'pain threshold'/exp OR 'nociception threshold' OR 'nociceptive threshold' OR 'pain threshold' OR 'threshold, pain')]

Table 1. Electronic search strategy for EMBASE.
